# Toward a Better Beauty Regimen: Reducing Potential EDC Exposures from Personal Care Products

**DOI:** 10.1289/ehp.124-A188

**Published:** 2016-10-01

**Authors:** Carrie Arnold

**Affiliations:** Carrie Arnold is a freelance science writer living in Virginia. Her work has appeared in *Scientific American*, *Discover*, *New Scientist*, *Smithsonian*, and more.

As a high school student in Salinas, California, Irene Vera was part of the Youth Community Council, a project of the University of California, Berkeley, Center for the Health Assessment of Mothers and Children of Salinas (CHAMACOS). This project aims to train local youth as environmental health leaders through research, education, and advocacy, and it was through the Youth Community Council that Vera learned about potential endocrine-disrupting chemicals in personal care products. She and fellow council members worked with Kim Harley, an environmental health scientist at UC Berkeley, to study how teen girls are exposed to these chemicals and steps they can take to lower their exposures.^1^

**Figure:**
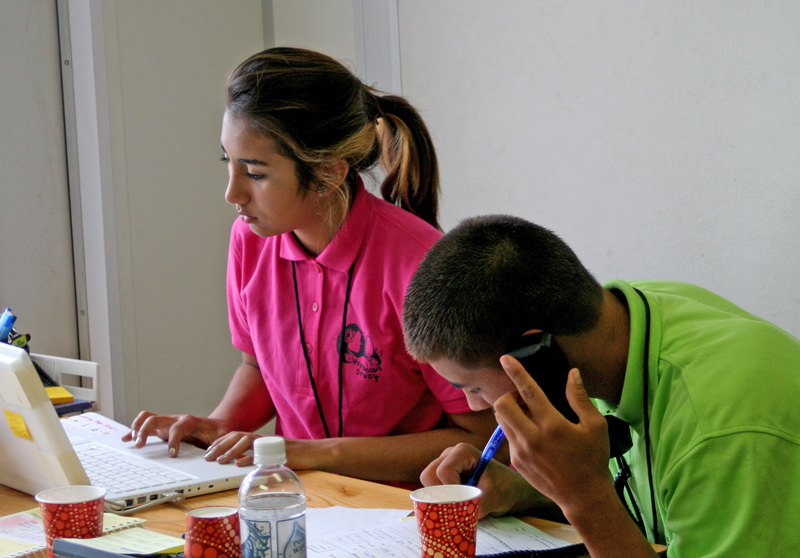
Irene Vera (left) and other members of the Youth Community Council served as research assistants on the HERMOSA study. © Kimberly Parra

The HERMOSA (Health and Environmental Research on Makeup of Salinas Adolescents) study measured the exposure of Latina teens to certain phthalates, parabens, triclosan, and benzophenone-3 in makeup and other personal care products (*hermosa* means “beautiful” in Spanish). These compounds have shown endocrine activity in animal and cell studies.^2^ ^,^ ^3^ ^,^ ^4^ ^,^ ^5^

Women are disproportionately exposed to phthalates and parabens because they use more personal care products on average than men.^6^ Teenage girls tend to use even more products than women, averaging 17 different products per day, compared with 12 for women.^7^

What remains unclear, however, is whether these exposures actually harm people. “We don’t know if there are long-term health effects of [these] chemicals, but we have reasons to be concerned about exposure to teenage girls, who often use a lot of these products during a time of rapid reproductive development,” Harley says.

It is also unclear how much of a person’s total exposure comes specifically from personal care products. Parabens, phthalates, benzophenone-3, and triclosan are widely used, says Ruthann Rudel, director of research at the Silent Spring Institute. “With chemicals that are so common,” she says, “it’s hard to know which sources influence exposure the most.” Rudel was not involved in the study.

Vera and the other teens on the Youth Community Council were involved with every aspect of the study, from recruiting participants to analyzing data. The students enrolled friends and classmates for a final sample size of 100 Latina adolescents from around the Salinas area. All the girls were educated on the potential risks of endocrine disruptors as a way to motivate both participation and compliance with the study protocol.

Girls who agreed to participate were provided with replacement personal care products and instructed to use these alternatives for 3 days. The replacement products were chosen on the basis of whether their ingredient lists included triclosan, BP-3, or parabens. Phthalates are not listed on ingredient lists, but they are often found in scented products. So the researchers avoided products that listed “fragrance” as an ingredient unless they were specifically labeled as phthalate free.^1^

Urine samples were collected at the beginning and end of the 3-day period. Analyses of urine samples showed that more than 90% of the HERMOSA participants had detectible levels of phthalates, parabens, and benzophenone-3 before they started using the replacement products, with most levels higher than average concentrations estimated for teens in the general U.S. population.^1^

After using the alternative products for 3 days, however, urinary concentrations of methyl and propyl paraben decreased by 43.9% and 45.4%, respectively, mono-ethyl phthalate decreased by 27.4%, and triclosan decreased by 35.7%. On the other hand, there were increases in concentrations of butyl and ethyl paraben, which were detected in about half the girls. The authors suggest that these chemicals might have been unintentional contaminants or unlabeled ingredients in replacement products, which they acknowledge they were unable to ensure were paraben free.^1^

These results indicate that consumer choices can affect exposures to the study compounds. “We were really pleased to see that after three days, [most] levels decreased by twenty-five to forty-five percent, on average,” Harley says. “When we started the project, we hadn’t seen any other studies that showed changing your personal care products and makeup would lower your levels of these [chemicals].”

Sheela Sathyanarayana, an environmental health scientist at the University of Washington, has high praise for how the study was carried out. “It was a very well-conducted study and an incredibly savvy approach to involve the study participants in their own research project. This only adds to the quality of the work,” says Sathyanarayana, who was not involved with the study.

Vera, now a sophomore at UC Santa Cruz, says the project sparked a desire to pursue a career in environmental law and policy. It also influenced her thinking on the personal care products she chooses. “I had never heard of these chemicals or looked at the labels on my personal care products until this project,” she says. But now, she adds, “I’ve switched out face, hair, and body products for … low-chemical replacements as a result of this work.”
